# The host cell side of latent HIV-1 infection

**DOI:** 10.18632/oncotarget.5101

**Published:** 2015-08-05

**Authors:** Lillian Seu, Olaf Kutsch

**Affiliations:** Department of Medicine, University of Alabama at Birmingham, Birmingham, Alabama, USA

**Keywords:** HIV-1, latency, pathogen- host cell interaction, kinome analysis

The ability of HIV-1 to establish an extremely stable latent viral reservoir in the CD4^+^ memory T cell population prevents viral eradication with the currently available antiretroviral drugs. A viral reservoir consisting of only 10_5_ latently HIV-1 infected T cells could take more than 60 years to dissipate, making natural eradication during the lifetime of a patient impossible [1]. To achieve viral eradication therapeutic intervention will be needed.

The fact that latent HIV-1 infection has mostly been described in the memory T cell population, which forms part of our lifelong immunity against pathogens, seems to justify the extraordinary stability of the viral reservoir. However, while immunity is lifelong, the lifespan of individual memory T cells is limited. There is no final consent on the life-span of memory T cells, but the higher range of estimates would suggest a half-life Δ_1/2_ = ~100 days for a CD4^+^ memory T cell, and shorter in HIV-1 patients, certainly not even in the range of the stability of the latent HIV-1 reservoir that has a calculated Δ_1/2_ = ~40 months [1]. To explain this discrepancy, studies have shown that latently infected T cells can undergo homeostatic proliferation in the absence of HIV-1 reactivation [2] as to maintain stability of the latent reservoir. HIV-1 integration into genes that promote clonal expansion of latently HIV-infected cells and slow the decay of the viral reservoir has also been described [3].

Viral reservoirs have been described in central memory T cells (T_CM_) and in effector memory T cells (T_EM_), and a more recent study described initial progressive reservoir contraction until a steady state in an extremely stable reservoir around a core of less-differentiated, stem cell-like memory CD4+ T cells (T_SCM_) was achieved [4]. However, no matter the exact nature of host cells of the latent HIV-1 reservoir, under the assumption that latent HIV-1 infection events are established in functional memory T cells, we should observe continuous and complete contraction of the reservoir as these T cells encounter their cognate antigen over time, as a cognate antigen induced recall response should then trigger HIV-1 reactivation. If latent HIV-1 infection were associated with functional memory T cells, to explain the reservoir stability, latent HIV-1 infection events would have to be exclusively established in memory T cells that recognize extremely rare antigens, or would have to be established in T cells that recognize HIV-1 specific antigens, an antigen resource that then would have to be completely eliminated by the initiation of ART.

A recent study by the Siliciano laboratory suggests a third possibility [5]. In Ho *et al*., the authors demonstrated that following *ex vivo* activation of resting T cells from HIV patients on ART a total of 1.7% of noninduced HIV-1 proviruses had completely intact genomes and LTR function and when reconstructed were replication competent. Most importantly, these noninduced viruses had no CpG methylation patterns that were suggestive of transcriptional silencing of the viral promoter. Thus, another explanation for the stability of the reservoir could be that the latent HIV-1 reservoir is associated with an unresponsive T cell phenotype. In Seu *et al*. [6] we could now demonstrate that host-cells of latent HIV-1 infection events were functionally altered in ways that are consistent with this idea.

Host cells of latent HIV-1 infection events exhibited a massively altered kinetic NF-κB response and interventions that induced T cell anergy either promoted latency establishment or stabilized latent infection events. Having already previously demonstrated that kinases can have a gate-keeper function in the control of latent HIV-1 infection [7], we used kinome array analysis of latently infected T cells to explore whether latently infected cells were phenotypically altered at the systems level. Indeed, latently HIV-1 infected T cells had a massively altered kinome signature when compared to uninfected control cells. Many changes were associated with pathways related to cell cycle, apoptosis and metabolism. Beyond providing insights into the complex interplay of the host cell with the virus that would stabilize latent HIV-1 infection, we could demonstrate that kinome array analysis can be used to identify drug targets to trigger HIV-1 reactivation using synergistically drug combinations consisting of a priming drug, that would restore cell responsiveness and a second, synergistic activator.

Our results emphasize that despite the high clinical relevance of latent HIV-1 infection, our understanding of the molecular mechanisms controlling latent HIV-1 infection still remains incomplete. Clearly, the stable phenotypic changes to the host-cells of latent infection events that we propose to control latent HIV-1 infection are a distinct possible, as functional impairment of antigen-specific T cells is a defining characteristic of many chronic infections, the classic example being up-regulation of PD-1 during chronic infection with lymphocytic choriomeningitis virus (LCMV).

If indeed host cells of latent infection events are rendered unresponsive, effective therapeutic strategies that seek to eradicate latent HIV-1 infection will first have to reprogram these host cells to become responsive to a subsequently administered HIV-1 reactivating stimulus. Our data provide little evidence that “magic bullet” approaches that hope to achieve full HIV-1 reactivation and subsequent viral eradication using a single drug have much promise to deliver a cure for HIV-1 infection.
